# Functional evaluation of constructed pseudo-endogenous microRNA-targeted myocardial ultrasound nanobubble

**DOI:** 10.3389/fmed.2023.1136304

**Published:** 2023-09-22

**Authors:** Ailifeire Ainiwan, Yuanyuan Wei, Jing Dou, Lingpeng Tang, Yuming Mu, Lina Guan

**Affiliations:** Department of Echocardiography, The First Affiliated Hospital of Xinjiang Medical University, Urumqi, China

**Keywords:** gene delivery, miRNA-1, targeted nanobubbles, ultrasound molecular imaging, pAd-AAV-9

## Abstract

**Background:**

Stem cell transplantation is one of the treatment methods for acute myocardial infarction (AMI). MicroRNA-1 contributes to the study of the essential mechanisms of stem cell transplantation for treating AMI by targeted regulating the myocardial microenvironment after stem cell transplantation at the post-transcriptional level. Thus, microRNA-1 participates in regulating the myocardial microenvironment after stem cell transplantation, a promising strategy for the Stem cell transplantation treatment of AMI. However, the naked microRNA-1 synthesized is extremely unstable and non-targeting, which can be rapidly degraded by circulating RNase. Herein, to safely and effectively targeted transport the naked microRNA-1 synthesized into myocardial tissue, we will construct pseudo-endogenous microRNA-targeted myocardial ultrasound nanobubble pAd-AAV-9/miRNA-1 NB and evaluate its characteristics, targeting, and function.

**Methods:**

The pAd-AAV-9/miRNA-1 gene complex was linked to nanobubble NBs by the “avidin-biotin bridging” method to prepare cardiomyocyte-targeted nanobubble pAd-AAV-9/miRNA-1 NB. The shape, particle size, dispersion, and stability of nanobubbles and the connection of pAd-AAV-9/miRNA-1 gene complex to nanobubble NB were observed. The virus loading efficiency was determined, and the myocardium-targeting imaging ability was evaluated using contrast-enhanced ultrasound imaging *in vivo*. The miRNA-1 expression level in myocardial tissue and other vital organs *ex vivo* of SD rats was considered by Q-PCR. Also, the cytotoxic effects were assessed.

**Results:**

The particle size of NBs was 504.02 ± 36.94 nm, and that of pAd-AAV-9/miRNA-1 NB was 568.00 ± 37.39 nm. The particle size and concentration of pAd-AAV-9/miRNA-1 NBs did not change significantly within 1 h at room temperature (*p* > 0.05). pAd-AAV-9/miRNA-1 NB had the highest viral load rate of 86.3 ± 2.2% (*p* < 0.05), and the optimum viral load was 5 μL (*p* < 0.05). pAd-AAV-9/miRNA-1 NB had good contrast-enhanced ultrasound imaging *in vivo*. Quantitative analysis of miRNA-1 expression levels in vital organs *ex vivo* of SD rats by Q-PCR showed that pAd-AAV-9/miRNA-1 NB targeted the myocardial tissue. Q-PCR indicated that the expression level of miRNA-1 in the myocardium of the pAd-AAV-9/miRNA-1 NB + UTMD group was significantly higher than that of the pAd-AAV-9/miRNA-1 NB group (*p* < 0.05). pAd-AAV-9/miRNA-1 NB had no cytotoxic effect on cardiomyocytes (*p* > 0.05).

**Conclusion:**

The pAd-AAV-9/miRNA-1 NB constructed in this study could carry naked miRNA-1 synthesized *in vitro* for targeted transport into myocardial tissue successfully and had sound contrast-enhanced imaging effects *in vivo*.

## 1. Introduction

MiRNAs are small non coding RNAs composed of 21–25 nucleotides, which regulate the myocardial microenvironment at the post transcriptional level (1, 2). Endogenous plasma miRNAs are released into the blood circulation in the form of microcapsules or exosomes (3), which can be protected from the influence of Ribonuclease (RNase) without being degraded, and exist in the circulation in a highly stable form (4). However, due to the characteristics of autologous secretion, it is difficult to obtain them. The naked miRNAs synthesized *in vitro* have the characteristics of high stability, conservatism, tissue specificity and timing. However, when the naked miRNAs synthesized are injected into the blood circulation *in vivo*, they will be rapidly degraded by RNase because of their non protective structure and non targeting (5), which will limit their role *in vivo*. How to protect the naked synthetic miRNAs injected into the blood circulation from being hydrolyzed by RNase, and how to safely transport them to the target area to perform their functions, is the most critical.

Ultrasound nanobubbles are also used as a new type of gene carrier transport tool, which can achieve targeted transport and transfection of target regions through surface binding with targeted molecular probes (6, 7). The capsid of recombinant adenovirus type 9 (pAd-AAV-9) is composed of capsid protein (8), which has no immunogenicity. It can not only serve as the protective layer of genes loaded and encapsulated, but also have a targeting effect on myocardial tissue (9). Therefore, this experiment simulates the existence form of endogenous miRNAs, the naked miRNA-1 synthesized *in vitro* was wraped by pAd-AAV-9 to construct pAd-AAV-9/miRNA-1 gene complex, which is provided by YunPark Biotech Co., Ltd. Then attach the pAd-AAV-9/miRNA-1 gene complex to the surface of ultrasound nanobubbles to construct pAd-AAV-9/miRNA-1 NB, measuring its physical and chemical properties, exploring its capacity of targeted transport gene loaded.

## 2. Materials and methods

### 2.1. Materials

PBS solution (Procell, Wuhan, China, P1020), DSPC (Ruixiang Biological Co., Ltd., Xi’an, China, HY-W040193), DSPE-PEG2000 (Ruixiang Biological Co., Ltd., Xi’an, China, PG2-BHDS-2 K), biotin (Bio, Xi’an, China, B4501), Octafluoropropane (C_3_F_8_, Air Liquide, Wuhan, China), nitrogen (N2, Xinyi, Urumqi, China), streptavidin (Sigma, United States, 85,878), biotinylated and FITC-labeled pAd-AAV-9/miRNA-1 (YunPark Biotech Co., Nanjing, China, 13,414 CE), biotinylated and FITC-labeled pAd-AAV-9 (YunPark Biotech Co., Nanjing, China, 133F0D4), H_9_C_2_ (Procell, Wuhan, China, CL-0089), DiI fluorescent dye (Biyun Biotechnology, Shanghai, China, 40718ES50), and CCK-8 (Iren, Japan, CKD4) were purchased. Trizol reagent (Servicebio, Wuhan, China, G3013), 2xSYBR Green qPCR Master Mix (None ROX) (Servicebio, Wuhan, China, G3320), Servicebio®RT First Strand cDNA Synthesis Kit (Servicebio, Wuhan, China, nG3330), Water Nuclease-Free (Servicebio, Wuhan, China, G4700), primer (Servicebio, Wuhan, China) were used in this study. Also, optical microscope (Leica, CTR6000, Germany), laser confocal microscope (Nikon, Japan), flow cytometer (Beckman, United States of America), and zeta analyzer (Malvern, United Kingdom), IVIS Imaging System (Caliper, MA, Hopkinton), Grinder low temperature type (Servicebio, Wuhan, China), Desktop high-speed refrigerated micro centrifuge (DragonLab, GuangZhou, China), CFX96 real-time fluorescent quantitative PCR system (Bio-rad) were used in this study.

### 2.2. Construction of NBs and pAd-AAV-9/miRNA-1 NBs

For constructing NBs, 20 mg DSPC, 18 mg DSPE-PEG2000, and 10 mg DSPE-PEG2000-biotin were dissolved in 1 mL of chloroform. Then, 532 μL/mL DSPC, 117 μL/mL DSPE-PEG2000, and 226 μL/mL DSPE-PEG2000-biotin were mixed to form a mother solution. The solvent in the mother liquor was removed at room temperature and under nitrogen flow. A thin layer of phospholipid film formed on the test tube wall. After 3 h of vacuum treatment to remove chloroform, 5 mL of Tris–HCl solution was added, and the phospholipid membrane was dissolved by mechanical shaking in a 65°C water bath. After filling the vial with C_3_F_8_, the vial was mechanically shaken for 30 s to obtain a bubble mixture and centrifuged at 300 rpm for 3 min. The NB in the lower layer was separated from the larger microbubbles in the upper layer. Finally, biotinylated NBs were successfully prepared and stored in the refrigerator at 4°C for later use. DiI-labeled NBs were prepared similarly, but 1 mg DiI was added to the initially formulated stock solution. The whole process progressed in a light-proof environment. pAd-AAV-9/miRNA-1 NBs were prepared by a biotin-avidin-biotin ligation method. The biotinylated NBs were washed with phosphate-buffered saline (PBS), incubated with avidin for 30 min, and centrifuged at 300 rpm for 3 min to remove free avidin. They were further incubated with different concentrations of the pAd-AAV-9/miRNA-1 gene complex at room temperature, centrifuged at 300 rpm for 3 min to remove excess biotinylated pAd-AAV-9/miRNA-1 gene complexes and generate pAd-AAV-9/miRNA-1 NBs, and placed in a 4°C freezer for later use.

### 2.3. Characterization of NBs and pAd-AAV-9/miRNA-1 NBs

pAd-AAV-9/miRNA-1 NB were observed under an optical microscope. The particle size of nanobubbles was measured using the zeta analyzer. The concentration was measured using the hemocytometer. For stability, the suspension was diluted with PBS solution (1:100) at room temperature to 1 mL, and the particle size and concentration were detected after 5, 10, 15, 30, 45, and 60 min. Laser confocal microscope and flow cytometry analysis were used to evaluate the nanobubble loading efficiency of the pAd-AAV-9/miRNA-1 gene complex.

### 2.4. *In vitro* contrast-enhanced ultrasound imaging

The self-made agarose gel imaging matrix model was cut into pieces and fixed on the plate. NBs, pAd-AAV-9/miRNA-1 NB (1 × 10^8^pfu/μ L), and SonoVue, each 100 μL, were used to make homemade models. An appropriate amount of couplant was applied on the side of the model, and an ultrasonic diagnostic apparatus (Vivid 6, Mindray, China) was used at a frequency of 14 MH z and contrast mode to observe NBs, pAd-AAV-9/miRNA-1 NB, and SonoVue (Bracco Suisse SA, Switzerland) *in vitro*. The contrast-enhanced images were intercepted at each time point of 5 s, 15 s, 30 s, 1 min, 2 min, and 3 min and imported into the software ImageJ. The nanobubble contrast imaging area was defined as the region of interest (ROI), using the dB value to represent the signal intensity of the contrast imaging region and quantitatively analyzing the signal intensity of NBs, pAd-AAV-9/miRNA-1 NB, and SonoVue at different time points in the contrast imaging region. The probe position and ultrasound parameters remained unchanged throughout.

### 2.5. *In vitro* cell viability assay

CCK-8 (Iren, Japan) was used to detect the cytotoxicity of pAd-AAV-9/miRNA-1 NBs on SD rat cardiomyocytes. The cardiomyocytes and different concentrations of pAd-AAV-9/miRNA-1 NBs were inoculated in a 96-well plate. After 48 h of incubation, CCK-8 solution was added to the experimental group, the positive control group, and the negative control group, with an amount of 10 μL per well, placed in an incubator for 2–4 h, and then labeled with an enzyme label. The instrument measured the absorbance of each well in the three groups at 450 nm, namely the OD value. The following formula was used to calculate the cell viability.


$$\mathrm{Cell}\;\mathrm{viability}\%=\left[\begin{array}{l} \left(\mathrm{experimental}\;\mathrm{group}-\mathrm{negative}\;\mathrm{control}\;\mathrm{group}\right)/\\ \left(\mathrm{positive}\;\mathrm{control}\;\mathrm{group}-\mathrm{negative}\;\mathrm{control}\;\mathrm{group}\right) \end{array}\right]\times 100\%\text{.}$$

Experimental group: SD rat cardiomyocyte-specific complete medium, CCK-8, and nanobubble solution.

Positive control group: SD rat cardiomyocyte-specific complete medium, CCK-8, without nanobubble solution.

Negative control group: CCK-8, without SD rat cardiomyocyte-specific complete medium and nanobubble solution.

### 2.6. *In vivo* contrast-enhanced ultrasound imaging

6 SD male rats, aged 7–8 weeks and weighing about 190–210 g, were provided by the Animal Experiment Center of Xinjiang Medical University, with the ethics review approval number (K202202-12). The rats were randomly divided into pAd-AAV-9/miRNA-1 NB group (*n* = 3) and NB group (*n* = 3). The small animals were anesthetized with 2.5% isoflurane gas (2 L/min) using a small-animal anesthesia machine, placed on a flat plate supine, and shaved to expose the chest. An appropriate amount of ultrasonic coupling agent was applied on the surface. Using an ultrasonic diagnostic machine (Vivid 6, Mindray, China), the center frequency of the probe (L13) was 14 MHz, and the long-axis section of the parasternal left ventricle of SD rats was displayed in the two-dimensional mode and then the contrast mode was entered. Further, 100 μL each of pAd-AAV-9/miRNA-1 NB (1 × 10^8^pfu/μ L) and NBs was injected through the tail vein at a concentration of about 1 × 10^7^/mL. The arrival of nanobubbles in the myocardial tissue was recorded within 180 s, following which the images of contrast imaging were intercepted at each time point of 5 s, 15 s, 30 s, 1 min, 5 min, 10 min, and 15 min. The probe position and ultrasound parameters remained unchanged throughout.

The image was imported into the software ImageJ. The nanobubble imaging area was defined as the ROI, and the signal intensity of the imaging area was represented by the dB value. The enhanced imaging effects of the two nanobubbles in SD rat myocardial tissue were evaluated by quantitatively analyzing the difference in the signal intensity (dB) of NBs and pAd-AAV-9/miRNA-1 NBs *in vivo* angiographic imaging areas at different time points.

### 2.7. pAd-AAV-9/miRNA-1 NB in the vital organs of rats

19 SD male rats, aged 7–8 weeks and weighing about 190–210 g, were randomly divided into pAd-AAV-9/miRNA-1 NB group (*n* = 9), NB group (*n* = 9), and control group (*n* = 1), and each group was randomly divided into three subgroups: time points 5 min (*n* = 3), 15 min (*n* = 3), and 35 min (*n* = 3). Then, 100 μL of Dil fluorescently labeled pAd-AAV-9/miRNA-1 NB (1 × 10^8^pfu/μ L) and NBs were injected through the tail vein, and the animals were sacrificed after 5 min, 15 min, and 35 min. The spleen, liver, and kidney *ex vivo* were arranged in order on a black cardboard, and the cardboard was placed in the IVIS imaging system of the small-animal imager. The parameters were set as DiI maximum excitation wavelength 549 nm and maximum emission wavelength 565 nm, and tissue fluorescence imaging was performed.

The image was acquired after exposure, and the built-in ROI quantitative analysis data tool of the imager IVIS was used. The signal intensity of the Dil uptake fluorescence ROI was represented by the dB value. The signal intensity of the Dil uptake of the fluorescent ROI in each isolated tissue in each group was obtained for comparative analysis.

### 2.8. Quantification of miRNA-1 expression levels in vital organs *ex vivo* of SD rats by Q-PCR

SD male rats, aged 7–8 weeks and weighing about 190–210 g, were randomly selected and divided into a 5 min group (*n* = 3), a 15 min group (*n* = 3), and a 35 min group (*n* = 3). The SD rats were anesthetized with 2.5% isoflurane gas (2 L/min) in a small animal anesthesia machine and then placed on a flat plate. Each group was injected with 100 μ L pAd-AAV-9/miRNA-1 NB (1 × 10^8^pfu/μ L) through the tail vein, and the corresponding 5, 15 and 35 min groups of rats were then sacrificed at 5, 15 and 35 min of blood circulation, and the *ex vivo* heart, kidney, spleen, lung, and liver of each SD rat in each group were placed in 5 clean centrifuge tubes.

PCR: Total RNA was extracted from each group of *ex vivo* heart, kidney, spleen, lung, and liver tissue samples using the Trizol reagent as per the manufacturer’s instructions. Nanodrop 2000 was used to detect RNA concentration and purity. 20 μL of total RNA from each sample was reverse transcribed using a Servicebio®RT First Strand cDNA Synthesis Kit, and real-time quantitative PCR (qPCR) reactions were performed using a CFX96 real-time fluorescent quantitative PCR system. 2x SYBR Green qPCR Master Mix (None ROX) was used for qPCR reactions with U6 as an internal reference gene. Relative gene expression was calculated using the 2-ΔΔCT method, and reactions were performed in triplicate to ensure accuracy. Primer sequences are shown in Supplementary Table S1.

### 2.9. Quantification of miRNA-1 expression levels in myocardial tissue *ex vivo* of SD rats by Q-PCR

Experimental grouping: 12 SD male rats, aged 7–8 weeks and weighing about 190–210 g, were randomly divided into the following 4 groups according to different intervention methods: ① NBs group (*n* = 3): NBs was injected through tail vein, ② pAd-AAV-9 NB group (*n* = 3): pAd-AAV-9 NB was injected via tail vein, ③ pAd-AAV-9/miRNA-1 NB group (*n* = 3): pAd-AAV-9/miRNA-1 NB was injected through tail vein, and ④ pAd-AAV-9/miRNA-1 NB + UTMD group (*n* = 3): pAd-AAV-9/miRNA-1 NB was injected through tail vein, and ultrasonic irradiation was performed.

Preparation of transfection solution: ① pAd-AAV-9 NB: 5 μL pAd-AAV-9 virus solution (1 × 10^8^pfu/μ L) was incubated with 100 μL NB, and ② pAd-AAV-9/miRNA-1 NB: 5 μL pAd-AAV-9/miRNA-1 virus solution (1 × 10^8^pfu/μ L) was incubated with 100 μL NB. The concentrations of pAd-AAV-9 and pAd-AAV-9/miRNA-1 virus solutions are determined according to the experimental result of pAd-AAV-9/miRNA-1 NB virus loading efficiency in this study.

Ultrasonic irradiation blasting conditions: connect probe (L13), signal generator and power amplifier, set mechanical index as 0.6, sound pressure as 0.6 MPa, duty cycle as 50%, and irradiation time as 120 s.

Operation steps: the SD rats were anesthetized with 2.5% isoflurane gas (2 L/min) in a small animal anesthesia machine and then placed on a flat plate. NBs group: 100 μL NBs was injected through the tail vein. pAd-AAV-9 NB group: 100 μL pAd-AAV-9 NB was injected through the tail vein. pAd-AAV-9/miRNA-1 NB group: 100 μL pAd-AAV-9/miRNA-1 NB was injected into the tail vein. The rats in each group were killed after 15 min of blood circulation, and the hearts were dissected and put into centrifuge tubes. pAd-AAV-9/miRNA-1 NB + UTMD group: The chest was exposed by shaving after anesthesia and an appropriate amount of ultrasound couplant was applied on the surface. First, 100 μL pAd-AAV-9/miRNA-1 NB was injected through the tail vein, and then the ultrasonic diagnostic machine (Vivid 6, Mindray, China) was used after slow injection. The central frequency of the probe (L13) was 14 MHz. The long-axis section of the parasternal left ventricle of SD rats was displayed in the two-dimensional mode, then switched the imaging mode to the contrast mode, used the set ultrasonic irradiation blasting conditions for irradiation for 120 s. After 15 min of blood circulation, the rats were killed and the heart were dissected and put into a centrifuge tube. The gun head and centrifuge tube are sterilized by moist heat, and there is no RNA enzyme.

Q-PCR: Total RNA was extracted from myocardial tissue in four samples using the Trizol reagent as per the manufacturer’s instructions. Nanodrop 2000 was used to detect RNA concentration and purity. 20 μL of total RNA from each sample was reverse transcribed using a Servicebio®RT First Strand cDNA Synthesis Kit, and real-time quantitative PCR (qPCR) reactions were performed using a CFX96 real-time fluorescent quantitative PCR system. 2x SYBR Green qPCR Master Mix (None ROX) was used for qPCR reactions with U6 as an internal reference gene. Relative gene expression was calculated using the 2-ΔΔCT method, and reactions were performed in triplicate to ensure accuracy. Primer sequences are shown in Supplementary Table S1.

### 2.10. Statistical analysis

Statistical analysis was performed using SPSS25.0 software. The measurement data conforming to the normal distribution were represented as “ $\bar{x}\pm s$ .” The unpaired independent-sample two-tail *t* test was used to compare the means of the two groups. The data analysis of multiple groups was performed by one-way analysis of variance (ANOVA) or repeated-measures multivariate ANOVA. The LSD method was used for pairwise comparison between the groups. Setting *α* = 0.05, *p* values <0.05 indicated statistically significant differences.

## 3. Results

### 3.1. Representation

**Figure 1:**
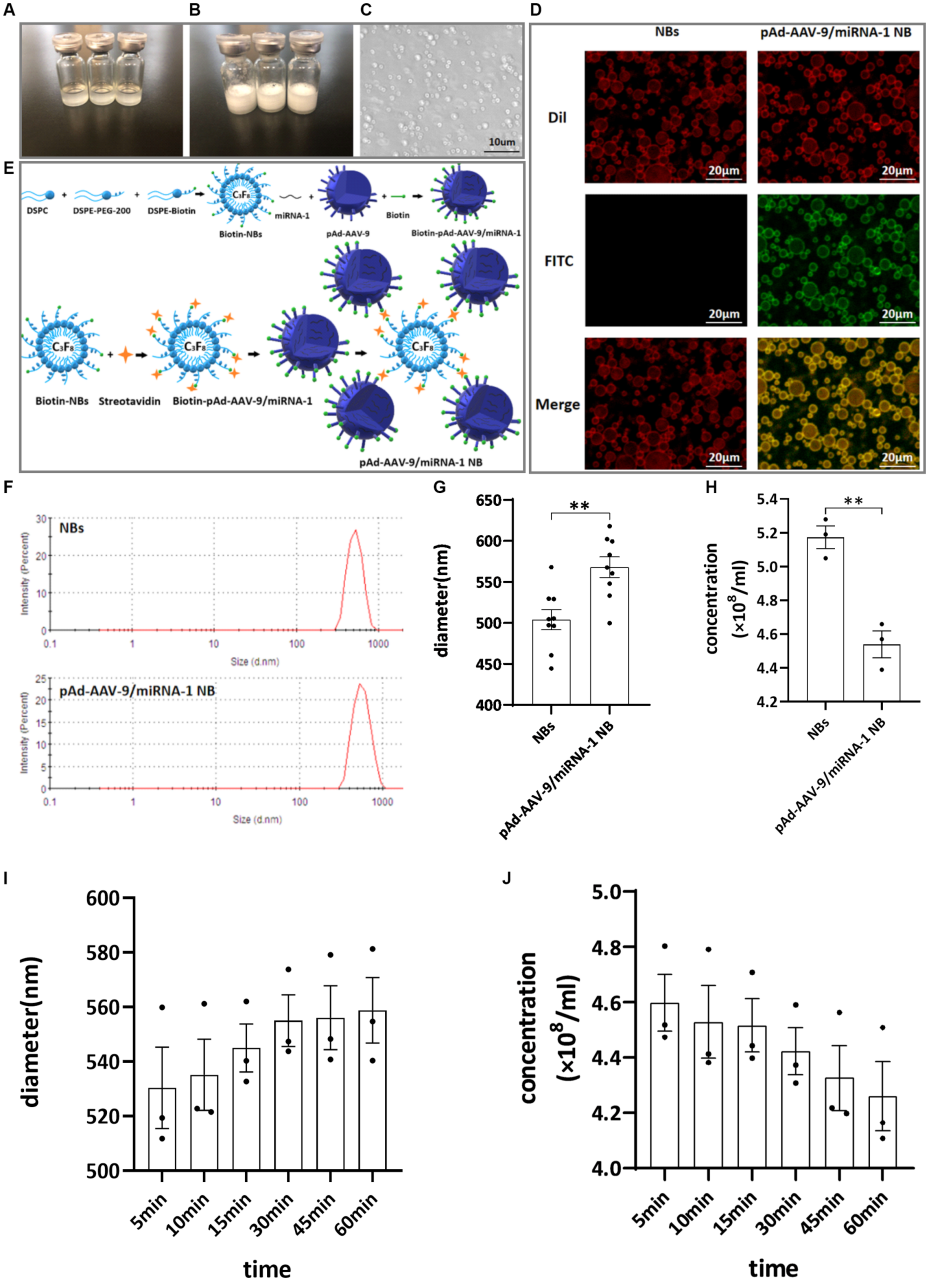
General characteristics. **(A)** Clear NB lipid. **(B)** After mechanical vibration. **(C)** Microscopic image of NBs. **(D)** Connection of NBs with pAd-AAV-9/miRNA-1 gene complex by laser confocal microscope. **(E)** Schematic diagram of pAd-AAV-9/miRNA-1 targeted NBs. **(F)** Particle size distribution representative images of NBs and pAd-AAV-9/miRNA-1 NB by zeta analyzer. **(G)** Quantitative analysis of the diameter of NBs and pAd-AAV-9/miRNA-1 NB by zeta analyzer, data: mean ± SEM, *p < 0.05, the experiments were run three times, *n* = 3 per group. **(H)** Quantitative analysis of the concentration of NBs and pAd-AAV-9/miRNA-1 NB by zeta analyzer, data: mean ± SEM, *p < 0.05, the experiments were run three times, *n* = 3 per group. Stability of pAd-AAV-9/miRNA-1 NB by zeta analyzer, the changes in diameter **(I)** and concentration **(J)** of pAd-AAV-9/miRNA-1 NB at different time points.

The nanobubble solution was a milky white mixed suspension. Divide into two layers after standing for 5–10 min at room temperature, The upper layer is a milky white nanobubble liquid, while the lower layer is a clear liquid. The morphology of the NBs was observed using an optical microscope. They were found to be spherical, uniformly dispersed, and regularly distributed, as shown in Figures 1A–C.

The biotin-avidin method was used to connect biotinylated nanobubble NBs with biotinylated pAd-AAV-9/miRNA-1gene complexes to construct targeted nanobubbles pAd-AAV-9/miRNA-1 NB, as shown in Figure 1E.

Under two different fluorescence excitation spectra (488 nm, 549 nm) were observed using a laser confocal microscope to detect the connection of NBs with pAd-AAV-9/miRNA-1 gene complexes. The NBs only labeled with Dil fluorescence were red under Dil excitation spectrum exposure while showed no color under FITC excitation spectrum exposure. The pAd-AAV-9/miRNA-1 NB labeled with both Dil fluorescence and FITC luorescence were red under Dil excitation spectrum exposure and green under FITC excitation spectrum exposure. Moreover, Targeting nanobubbles pAd-AAV-9/miRNA-1 NB were yellow under the fusion of two different excitation spectra, while the NBs were still colored red, which showed that the biotinylated pAd-AAV-9/miRNA-1 gene complex were successfully linked to the biotinylated NBs, as shown in Figure 1D.

The particle size of NBs was (504.02 ± 36.94 nm), the PDI was (0.24 ± 0.92), and the concentration was (5.17 ± 0.11) × 10^8^/mL. The particle size of pAd-AAV-9/miRNA-1 NB was (568.00 ± 37.39 nm), the PDI was (0.17 ± 0.52), and the concentration was (4.54 ± 0.13) × 10^8^ /mL, as shown in Figures 1F–H. As shown in Table 1, although pAd-AAV-9/miRNA-1 NB has a larger particle size and lower concentration compared to NBs, the particle size of pAd-AAV-9/miRNA-1 NB is within the nanoscale range with a concentration of 10^8^ml, indicating that targeted nanobubbles pAd-AAV-9/miRNA-1 NB were successfully manufactured. Within 1 h of construction, the particle size and concentration of pAd-AAV-9/miRNA-1 NB did not change significantly, indicating that pAd-AAV-9/miRNA-1 NB has good stability, as shown in Figures 1I,J and Table 2.

**Table 1:** Diameter, PDI, and concentration NBs and pAd-AAV-9/miRNA-1 NB.

Index	Nanobubbles		*t*	*p*
	NBs	pAd-AAV-9/miRNA-1 NB		
Diameter (nm)	504.02 ± 36.94	568.00 ± 37.39^*^	−3.651	0.002
PDI	0.24 ± 0.92	0.17 ± 0.52	3.205	0.002
Concentration (×10^8^/mL)	5.17 ± 0.11	4.54 ± 0.13^*^	−6.101	0.004

^*^p < 0.05.

**Table 2:** Quantitative analysis of diameter and concentration of pAd-AAV-9/miRNA-1 NB at different time points.

Time (min)	Index	
	Diameter (nm)	Concentration (10^8^/mL)
5	530.36 ± 25.85	4.60 ± 17.88
10	535.16 ± 22.55	4.53 ± 22.80
15	545.00 ± 15.20	452 ± 16.75
30	555.00 ± 16.38	4.42 ± 14.80
45	556.06 ± 20.29	4.33 ± 20.46
60	558.80 ± 20.75	4.26 ± 21.71
*F*	1.008	1.358
*P*	0.454	0.304

### 3.2. pAd-AAV-9/miRNA-1 NB virus loading efficiency

**Figure 2:**
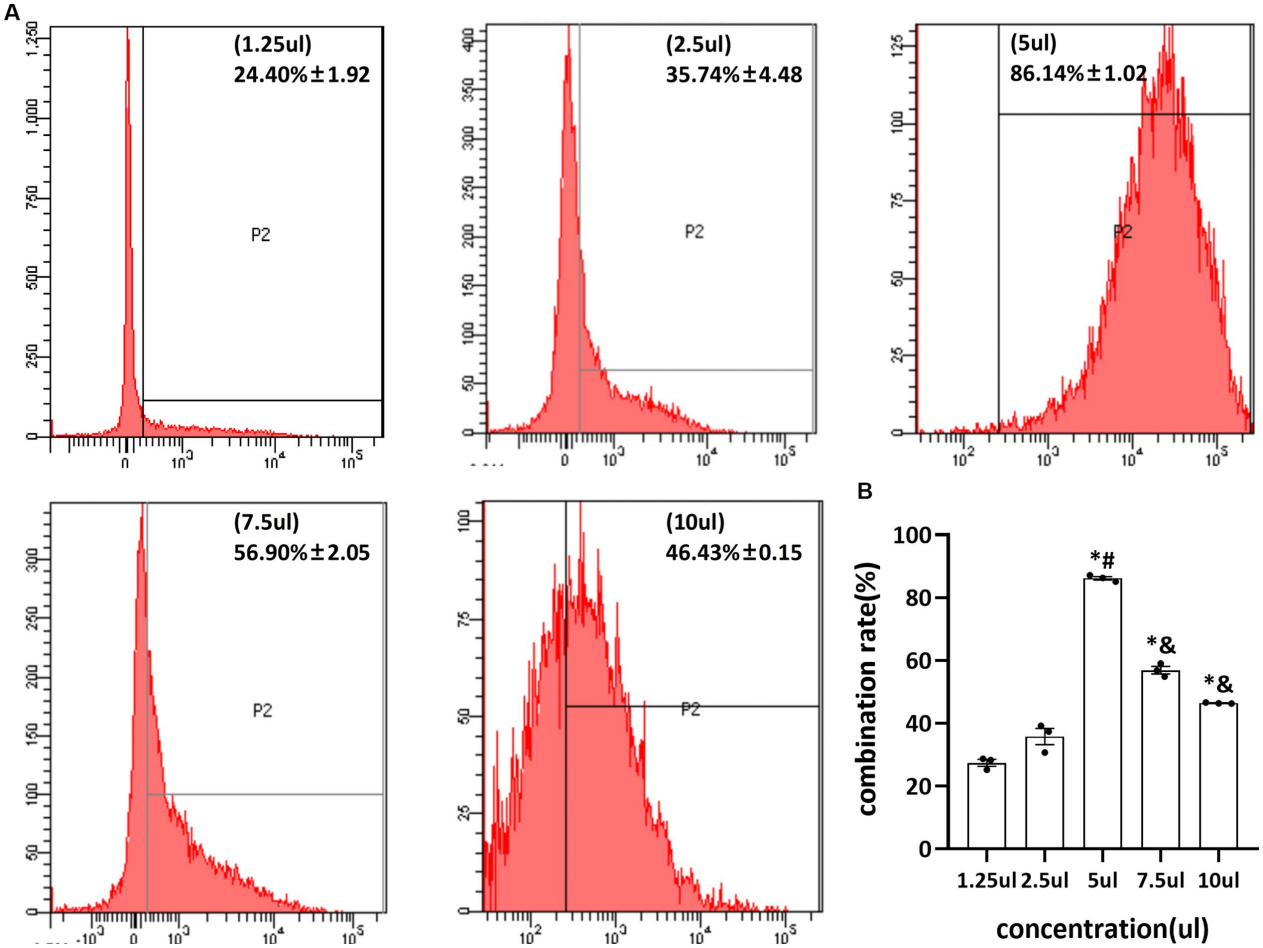
Virus loading efficiency of pAd-AAV-9/miRNA-1 NB by Flow cytometry. **(A)** Representative image of pAd-AAV-9/miRNA-1 NB virus loading efficiency. **(B)** Quantitative analysis of the pAd-AAV-9/miRNA-1 NB virus loading rate, data: mean ± SEM, ^*^p < 0.05 compared with the 1.25-μL group, ^#^*p* < 0.05 compared with the 2.5-μL group, ^&^*p* < 0.05 compared with the 5-μL group, the experiments were run three times, *n* = 3 per group.

Different concentrations of pAd-AAV-9/miRNA-1 gene complexes were incubated with 100 μL of NBs. The flow cytometry detected pAd-AAV-9/miRNA-1 NB virus loading efficiency increased first and then decreased with the increase of pAd-AAV-9/miRNA-1 gene complex dose. When the dosage of pAd-AAV-9/miRNA-1 gene complex is 5 μL, the viral loading rate of nanobubbles is the highest. However, with an increase in virus dose, the virus loading efficiency decreases, and the difference is statistically significant compared to 7.5 μL and 10 μL, as shown in Figure 2 and Table 3. Thus, the highest virus loading rate was (86.14 ± 1.02)%, and the optimum virus loading amount was 5 μL (*p* < 0.05). Determining the optimal viral load of pAd-AAV-9/miRNA-1 NB will be more conducive to its targeting performance, and save the amount of pAd-AAV-9/miRNA-1gene complex.

**Table 3:** Quantitative analysis of pAd-AAV-9/miRNA-1 NB virus load efficiency.

Concentration (μL)	Load efficiency (%)
1.25	24.4 ± 1.92
2.5	35.74 ± 4.48
5	86.14 ± 1.02 ^*#^
7.5	56.90 ± 2.05 ^*&^
10	46.43 ± 0.15 ^*&^
*F*	269.19
*p*	<0.05

^*^p < 0.05 compared with the 1.25-μL group, ^#^*p* < 0.05 compared with the 2.5-μL group, ^&^*p* < 0.05 compared with the 5-μL group.

### 3.3. Contrast ultrasound imaging *in vitro*

**Figure 3:**
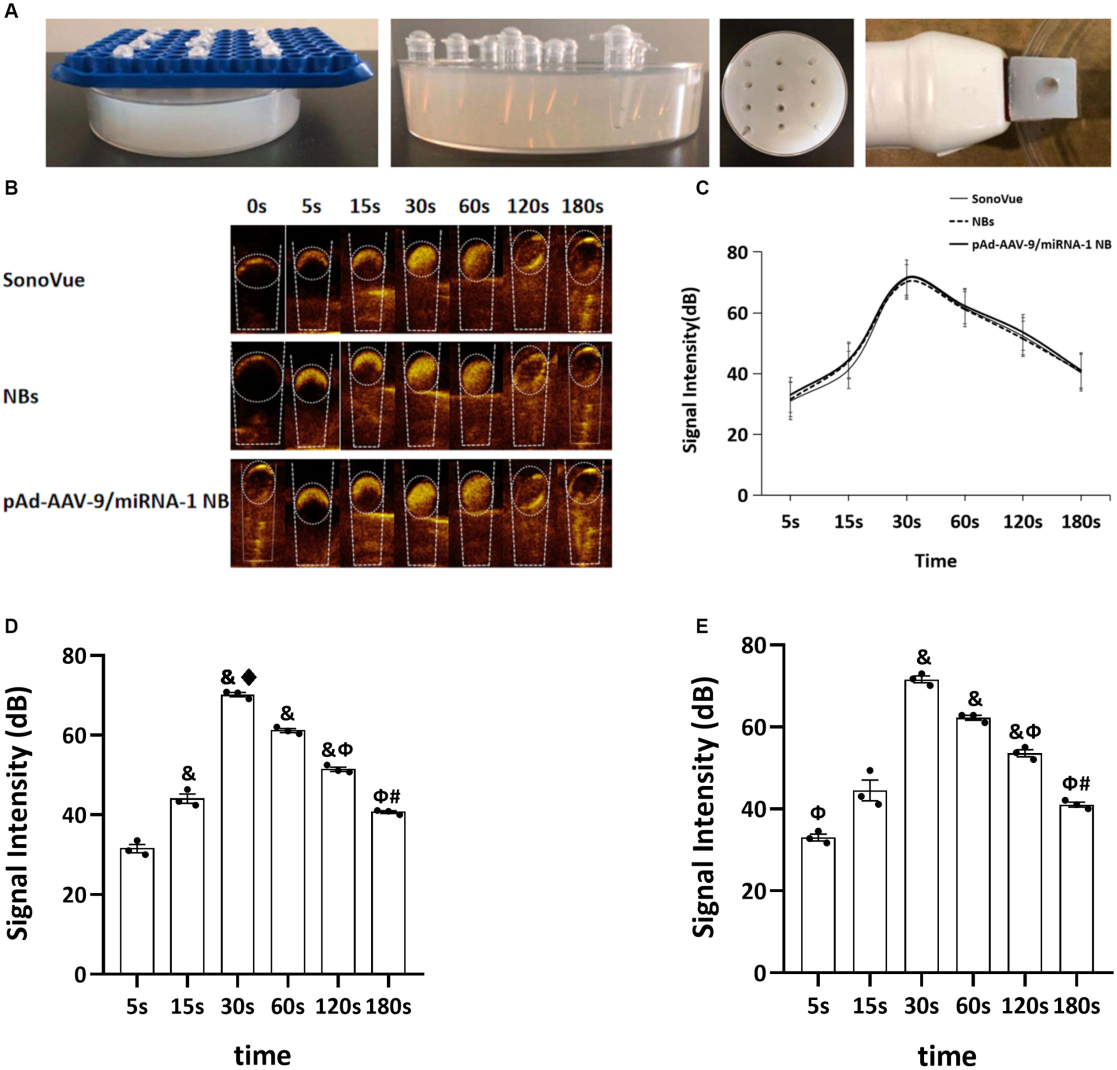
Contrast-enhanced ultrasound imaging *in vitro*. **(A)** Preparation process diagram of ultrasonic imaging in an agarose gel matrix model. **(B)** Contrast-enhanced ultrasound imaging of SonoVue, NBs, and pAd-AAV-9/miRNA-1 NB *in vitro*. **(C)** Quantitative comparative analysis of the ultrasound contrast time-signal intensity curves between SonoVue, NB, and pAd-AAV-9/miRNA-1 NB groups. **(D)** Quantitative analysis of ultrasound contrast-enhanced imaging signal intensity difference in the NB group at different time points. **(E)** Quantitative analysis of ultrasound contrast-enhanced imaging signal intensity difference in the pAd-AAV-9/miRNA-1 NB group at different time points. Data: mean ± SEM, ^&^*p* < 0.05 compared with the 5 s group, ^♦^*p* < 0.05 compared with the 15 s group, ^Φ^*p* < 0.05 compared with the 30 s group, ^#^*p* < 0.05 compared with the 60 s group, the experiments were run three times, *n* = 3 per group.

After the injection of SV, NBs, and pAd-AAV-9/miRNA-1 NB into the sample wells of the model, rapid development began after 5 s *in vitro*, reached the peak intensity after 30 s, and lasted for more than 3 min, as shown in Figures 3A,B. The signal intensity of the 3 groups showed a trend of increasing first and then gradually decreasing and the peak contrast signal intensity (dB) of SV, NBs and pAd-AAV-9/miRNA-1 NB was 71.20 ± 2.85 (dB), 70.19 ± 0.93 (dB), and 71.59 ± 1.44 (dB), respectively. No statistically significant difference (*p* > 0.05) was found in the contrast signal intensity (dB) between the 3 groups at different time points. In addition, the peak signal intensity of NBs and pAd-AAV-9/miRNA-1 NB showed statistical differences (*p* < 0.001) compared to the contrast signal intensity at other time points within their respective groups. It indicates that both self-made nanobubbles have good *in vitro* contrast enhanced imaging effects, as shown in Figures 3C–E and Table 4.

**Table 4:** Quantitative analysis of the signal intensity of contrast-enhanced imaging in SonoVue and two kinds of nanobubbles at different time points *in vitro.*

Time (s)	Signal intensity (dB)			*F*	*p*
	SovoVue	NBs	pAd-AAV-9/miRNA-1 NB		
5	30.97 ± 3.07	31.55 ± 1.80	33.00 ± 1.46	0.491	0.644
15	41.25 ± 1.29	44.04 ± 2.02^&^	44.49 ± 4.34	1,337	0.359
30	71.20 ± 2.85	70.19 ± 0.93^& ♦^	71.59 ± 1.44 ^&^	0.338	0.732
60	61.55 ± 1.40	61.13 ± 0.84^&^	62.23 ± 1.06 ^&^	0.661	0.565
120	52.29 ± 1.34	51.45 ± 0.93^& Ф^	53.58 ± 1.48 ^& Ф^	1.524	0.322
180	40.27 ± 0.98	40.67 ± 0.58^Ф#^	41.05 ± 1.04 ^Ф#^	0.398	0.696
F	321.28	351.98	133.86		
*p*	*p* < 0.001	*p* < 0.001	*p* < 0.001		

^&^*p* < 0.05 compared with the 5 s group, ^♦^*p* < 0.05 compared with the 15 s group, ^Φ^*p* < 0.05 compared with the 30 s group, ^#^*p* < 0.05 compared with the 60 s group.

### 3.4. *In vivo* myocardium-targeted ultrasound imaging

**Figure 4:**
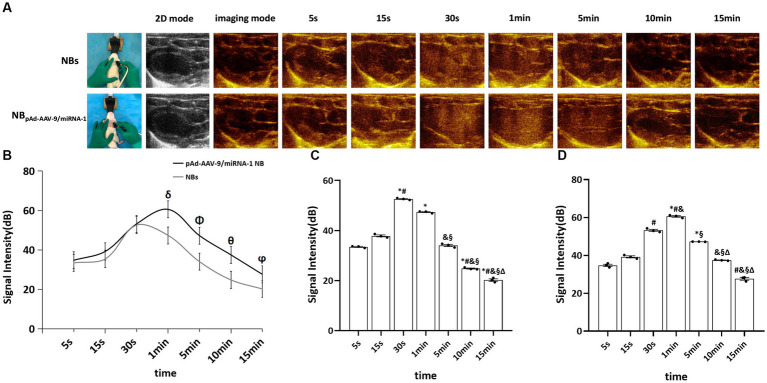
Myocardial targeted contrast-enhanced ultrasound imaging *in vivo.* **(A)** Contrast-enhanced ultrasound imaging of the heart after injection of NBs or pAd-AAV-9/miRNA-1 NB at different time points. **(B)** Quantitative comparative analysis of the ultrasound contrast time-signal intensity curves between the NB and pAd-AAV-9/miRNA-1 NB groups. **(C)** Quantitative analysis of ultrasound contrast-enhanced signal imaging intensity difference in the NB group. **(D)** Quantitative analysis of ultrasound contrast-enhanced signal imaging intensity difference in the pAd-AAV-9/miRNA-1 NB group at different time points. Data: mean ± SEM, ^δ^*p* < 0.05 compared with the signal intensity of NBs after 1 min, ^Φ^*p* < 0.05 compared with the signal intensity of NBs after 5 min, ^θ^*p* < 0.05 compared with the signal intensity of NBs after 10 min, ^ϕ^*p* < 0.05 compared with the signal intensity of NBs after 15 min, ^*^p < 0.05 compared with the signal intensity after 5 s, ^#^*p* < 0.05 compared with the signal intensity after 15 s, ^&^*p* < 0.05 compared the signal intensity after 30 s, ^§^*p* < 0.05 compared with the signal intensity after 1 min, ^Δ^ *p* < 0.05 compared with the signal intensity after 5 min, the experiments were run three times, *n* = 3 per group.

NB and pAd-AAV-9/miRNA-1 NB groups were injected with nanobubbles through the tail vein of SD rats. Both could initiate cardiac imaging rapidly *in vivo* after 5 s, as shown in Figure 4A. ROI quantitative comparison analysis was performed on the signal intensity of cardiac contrast ultrasound imaging. The NB group reached the peak imaging intensity in 30 s; the peak contrast signal intensity was 52.59 ± 0.38 (dB) and continued to increase for 5 min. The pAd-AAV-9/miRNA-1 NB group reached the peak imaging intensity in 1 min; the peak contrast signal intensity was 60.56 ± 0.74 (dB), and the imaging was continuously enhanced for more than 15 min (*P*<0.05). There were statistical differences (*p* < 0.05) between the peak imaging intensity and enhancement imaging duration of NB and pAd-AAV-9/miRNA-1 NB groups, indicating that the pAd AAV-9/miRNA-1 NB constructed in this study can target and enhance the development of the heart successfully, as shown in Figures 4B–D and Table 5.

**Table 5:** Quantitative analysis of the signal intensity of myocardium-targeted ultrasonic imaging in two nanobubbles at different time points *in vivo.*

Time	Signal intensity (dB)		*F*	*p*
	NBs	pAd-AAV-9/miRNA-1 NB		
5 s	33.44 ± 0.38	34.75 ± 1.06	8.622	0.099
15 s	37.84 ± 0.69	39.22 ± 0.95	6.147	0.131
30 s	52.59 ± 0.38^*#^	53.18 ± 0.96^#^	1.68	0.324
1 min	47.34 ± 0.37^*^	60.56 ± 0.74^δ*#&^	483.628	0.002
5 min	34.02 ± 0.75^&§^	47.24 ± 0.05^Φ*§^	824.958	0.001
10 min	24.78 ± 0.39 ^*# &§^	37.46 ± 0.30^θ &§Δ^	12971.77	0.001
15 min	20.17 ± 0.84 ^*# &§Δ^	27.59 ± 1.17 ^ϕ # &§Δ^	522.703	0.002
*F*	1465.128	628.935		
*p*	<0.001	<0.001		

^δ^*p* < 0.05 compared with the signal intensity of NBs after 1 min, ^Φ^*p* < 0.05 compared with the signal intensity of NBs after 5 min, ^θ^*p* < 0.05 compared with the signal intensity of NBs after 10 min, ^ϕ^*p* < 0.05 compared with the signal intensity of NBs after 15 min, ^*^p < 0.05 compared with the signal intensity after 5 s, ^#^*p* < 0.05 compared with the signal intensity after 15 s, ^&^*p* < 0.05 compared the signal intensity after 30 s, ^§^*p* < 0.05 compared with the signal intensity after 1 min, ^Δ^*p*< 0.05 compared with the signal intensity after 5 min.

### 3.5. pAd-AAV-9/miRNA-1 NB distribution in vital organs of rats

**Figure 5:**
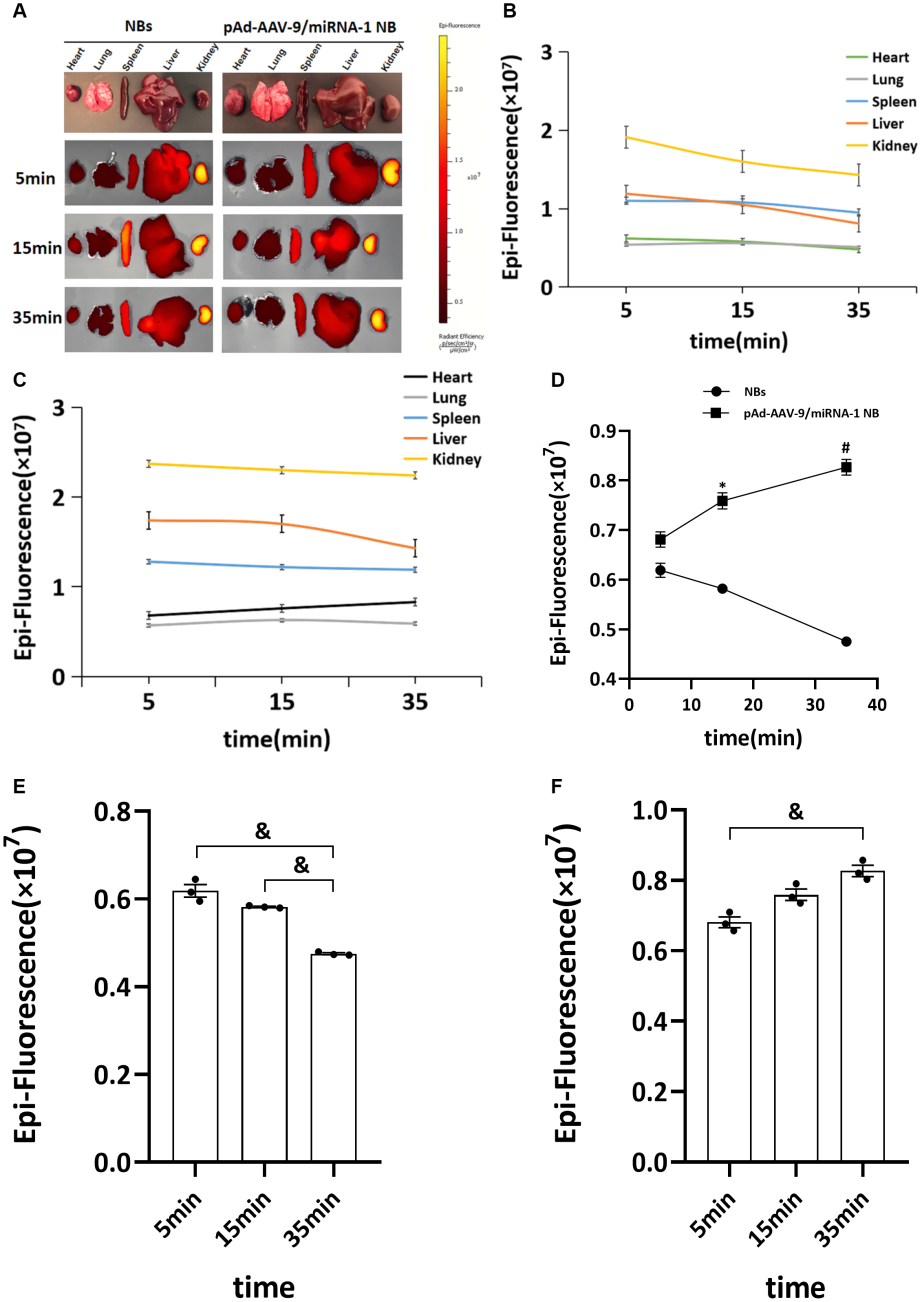
Biodistribution of NBs and pAd-AAV-9/miRNA-1 NB in SD rats by IVIS Imaging System. **(A)** *Ex vivo* fluorescence imaging of major organs of SD rats after the injection of NBs or pAd-AAV-9/miRNA-1 NB at different time points. **(B)** NB ROI analysis of fluorescence intensity of DiI uptake in different organs over time. **(C)** pAd-AAV-9/miRNA-1 NB ROI analysis of fluorescence intensity of DiI uptake in different organs over time. **(D)** Quantitative comparative analysis of the tendency of ROI fluorescence intensity between NBs and pAd-AAV-9/miRNA-1 NB in the heart after 5, 15, and 35 min. **(E)** Quantitative analysis of ROI fluorescence intensity of DiI uptake in the heart in the NB group after 5, 15, and 35 min. **(F)** Quantitative analysis of ROI fluorescence intensity of DiI uptake in the heart in the pAd-AAV-9/miRNA-1 NB group after 5, 15, and 35 min. Data: mean ± SEM, ^*^p < 0.05 compared with the fluorescence intensity of NBs after 15 min, ^#^*p* < 0.05 compared with the fluorescence intensity of NBs after 35 min, ^&^*p* < 0.05, the experiments were run three times, *n* = 3 per group.

To further confirm pAd-AAV-9/miRNA-1 NB targeting to myocardial tissue, we performed tissue fluorescence imaging on important organs such as heart, lung, spleen, liver, and kidney *ex vivo* after the injection of Dil fluorescently labeled NBs and pAd-AAV-9/miRNA-1 NB to two groups of SD rats via the tail vein, the results showed that Dil was detected in all important organs of the both NB group and pAd-AAV-9/miRNA-1 NB group. In the NBs group, the Dil uptake fluorescence signal intensity in the heart, lung, spleen, liver, and kidney attenuated over time, but he expression level of Dil uptake fluorescence signal intensity in the spleen and kidney was relatively high within 35 min, as shown in Figures 5A–C. In the pAd-AAV-9/miRNA-1 NB group, the Dil uptake fluorescence signal intensity in the lungs, spleen, liver, and kidneys attenuated over time, while the Dil uptake fluorescence signal intensity in the heart gradually increased from 5 min (6.81 ± 2.7 × 10^6^) to 35 min (8.27 ± 2.8 × 10^6^) (*p <* 0.05). However, the expression level of Dil uptake fluorescence signal intensity in the spleen and kidney was relatively high within 35 min, while the expression level of Dil uptake fluorescence signal intensity in the heart was relatively low within 35 min, as shown in Figures 5A–F and Table 6.

**Table 6:** Quantitative comparative analysis of the Dil uptake fluorescence signal intensity ROI between NBs and pAd-AAV-9/miRNA-1 NB in the heart at different time points.

Nanobubbles	Fluorescence signal intensity ROI (×10 ^6^)			*F*	*p*
	5 min	15 min	35 min		
NBs	6.19 ± 2.75	7.59 ± 2.82	8.27 ± 2.80	68.986	0.001
pAd-AAV-9/miRNA-1 NB	6.81 ± 2.7	5.82 ± 0.28^*^	4.75 ± 0.38^#^	17.086	0.011
*F*	5.896	121.617	609.420		
*p*	0.136	0.008	0.002		

^*^p < 0.05 compared with the fluorescence intensity of NBs after 15 min, ^#^*p* < 0.05 compared with the fluorescence intensity of NBs after 35 min.

### 3.6. Validation miRNA-1 in SD rat *ex vivo* vital organs by Q-PCR

**Figure 6:**
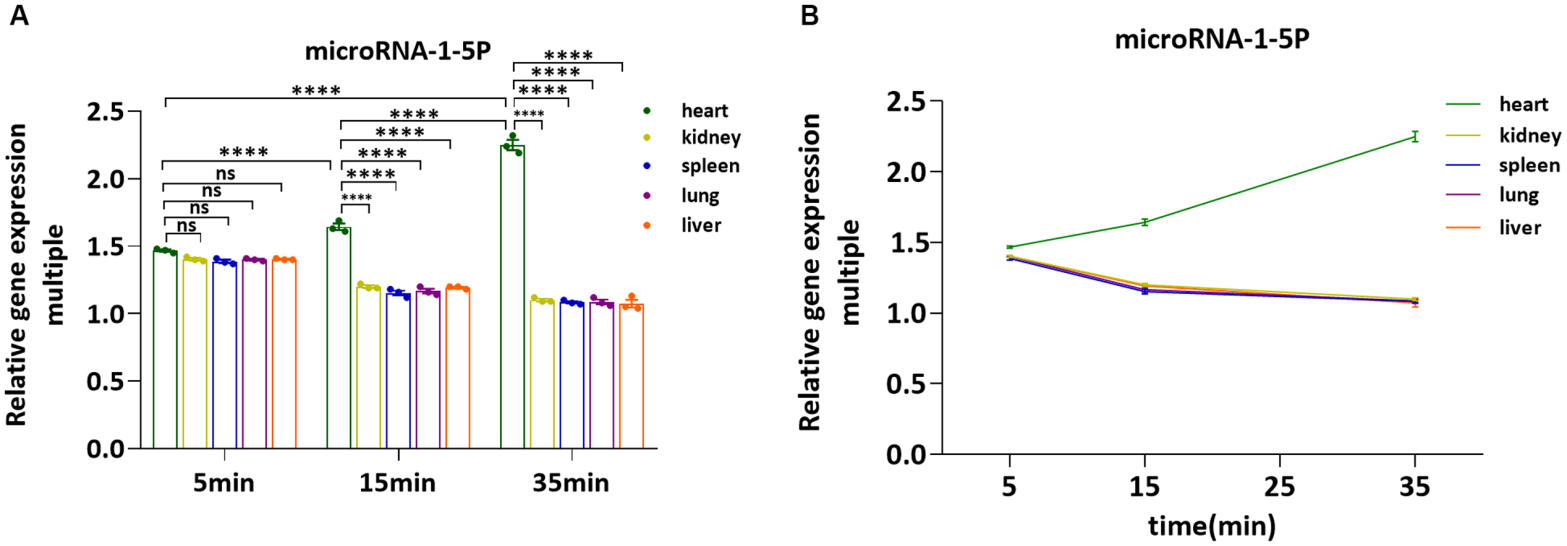
Fluorescence quantitative PCR validation of the expression levels of miRNA-1 in vital organs *ex vivo.* **(A)** Comparative analysis of the expression levels of miRNA-1in heart, kidney, spleen, lung, and liver of SD rats at different time points. **(B)** Analysis of the expression levels of miRNA-1 in different organs of SD rats over time. Data: mean ± SEM, ****p < 0.0001, the experiments were run three times, *n* = 3 per group.

After injection of NB or pAd-AAV-9/miRNA-1 NB at different time points, the fluorescence imaging results of the vital *ex vivo* organs of SD rats showed that the Dil uptake fluorescence signal intensity in the lung, spleen, liver, and kidney attenuated over time, but expression level of Dil uptake fluorescence signal intensity in the spleen and kidney was relatively high within 35 min. Thus, to confirm the introduction of miRNA-1 into the myocardial tissue via pAd-AAV-9/miRNA-1 NB, we evaluated the expression levels of miRNA-1 in different *ex vivo* organs of SD rats at different time points. Q-PCR results showed that at the 5-min time point, miRNA-1 expression was detected in all organs, but there was no statistically significant difference in the expression level of miRNA-1 between different organs, as shown in Figure 6A. Over time, the expression level of miRNA-1 in the heart continues to increase (****p < 0.0001), while its expression level gradually decreases in other organs, as shown in Figures 6A,B. Quantitative analysis showed that the expression levels of miRNA-1 in the heart were higher than those in other organs at both 15 and 35 min time points (****p < 0.0001), as shown in Figure 6A.

### 3.7. Validation miRNA-1 in SD rat *ex vivo* myocardial tissue by Q-PCR

**Figure 7:**
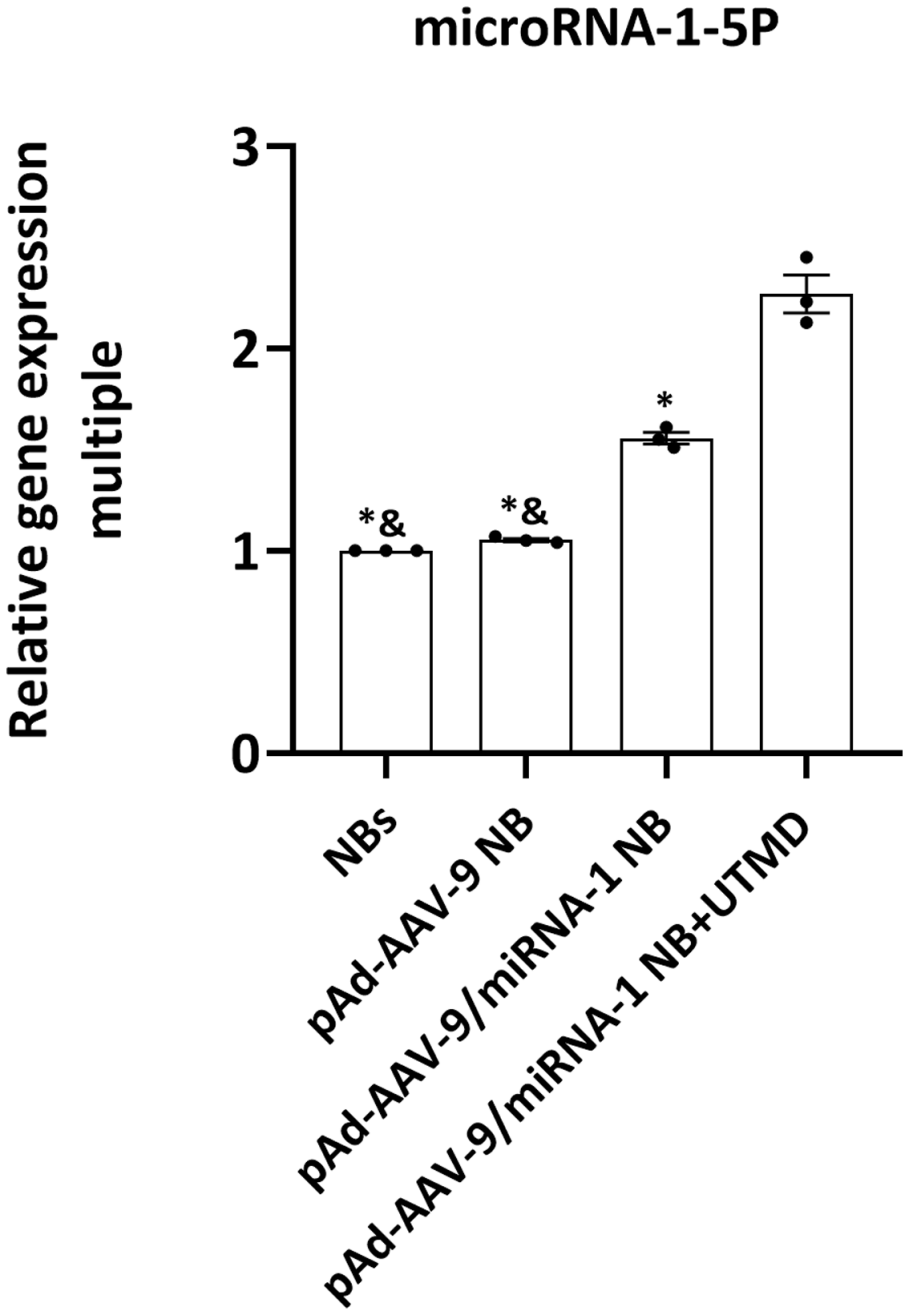
Fluorescence quantitative PCR validation of the expression levels of miRNA-1 of different groups in heart *ex vivo*. Data: mean ± SEM, ^&^*p* < 0.05 compared with the pAd-AAV-9/miRNA-1 NB group, ^*^p < 0.05 compared with the pAd-AVV-9/miRNA-1 NB + UTMD group, the experiments were run three times, *n* = 3 per group.

In order to further evaluate the ability of pAd-AAV-9/miRNA-1 NB to carry miRNA-1 into myocardium, we evaluated the expression level of miRNA-1 in the myocardial tissue *ex vivo* of SD rats. Q-PCR results showed that compared with the control, the expression of miRNA-1 was detected in the myocardium of pAd-AAV-9/miRNA-1 NB group and pAd-AAV-9/miRNA-1 NB + UTMD group; More importantly, the expression level of miRNA-1 in myocardium of pAd-AAV-9/miRNA-1 NB + UTMD group was significantly higher than that of pAd-AAV-9/miRNA-1 NB group (*p* < 0.05), as shown in Figure 7. These results indicate that the pAd-AAV-9/miRNA-1 NB can safely transport miRNA-1 into myocardial tissue.

### 3.8. *In vitro* cell viability assay

**Figure 8:**
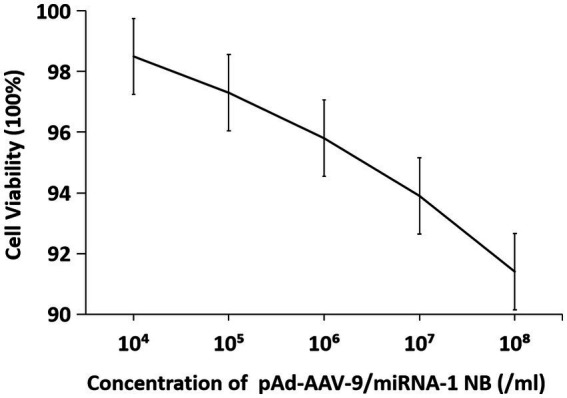
Cytotoxic analysis of pAd-AAV-9/miRNA-1 NB *in vitro*.

The cell viability *in vitro* of SD rat cardiomyocytes at different concentrations is shown in Figure 8 and Table 7. As the concentration of nanobubbles increases, the viability of SD rat cardiomyocytes in the logarithmic phase of growth gradually decreases. pAd-AVV-9/miRNA-1 NB concentration from 1 × 10^4^/ml increased to 1 × 10^8^/ml, the cell viability was 98.5 ± 2.3%, 97.3 ± 1.4%, 95.8 ± 1.3%, 93.9 ± 1.5%, and 91.4 ± 2.3%, respectively. However, even if pAd-AVV-9/miRNA-1 NB is at its highest concentration(1 × 10^8^/ml), it still exhibits cell activity of no less than 90%, and compared to the lowest concentration(1 × 10^4^/ml), there was no statistically significant difference in cell activity between the two concentrations(*p* > 0.05). It indicates that the pAd-AVV-9/miRNA-1 prepared in this experiment did not significantly damage cell activity, and has good biological safety performance.

**Table 7:** Quantitative evaluation of the effects of different concentrations of pAd-AAV-9/miRNA-1 NB on cell viability.

Concentration (/mL)	Cell viability (%)
10^4^	98.5 ± 0.16
10^5^	97.3 ± 0.13
10^6^	95.8 ± 0.12
10^7^	93.9 ± 0.14
10^8^	91.4 ± 0.08
*F*	0.409
*p*	0.801

## 4. Discussion

MicroRNAs (miRNAs) are a class of small noncoding RNAs that can regulate the remodeling process after myocardial infarction by regulating the myocardial microenvironment (10). When naked miRNA-1 synthesized *in vitro* is directly injected into the circulation, it is easily hydrolyzed by nucleases. In this study, by imitating the existence of endogenous miRNAs in the circulation *in vivo*, pAd-AAV-9 was used to encapsulate the naked miRNA-1 synthesized, and a targeted functional vector pAd-AAV-9/miRNA-1 carrying miRNA-1 was successfully assembled and constructed.

Previous studies have shown that the administration route of AAV vector is direct injection into the myocardium, which mediates local gene transfection. However, this method is invasive and the site of gene transfection is limited. Ultrasonic nanobubbles can achieve minimally invasive gene delivery at the cellular level by carrying targeted molecular probes on the surface. Therefore, in this study, pAd-AAV-9/miRNA-1 was attached to the surface of ultrasound nanobubbles, mediated by recombinant type 9 adenovirus and carried by lipid ultrasound nanobubbles, constituting a novel gene delivery system pAd-AAV-9/miRNA-1 NB, which targeted the myocardium and achieved precise, targeted, efficient, and minimally invasive gene transfection, thus avoiding the insufficiency of local-range gene transfection therapy.

pAd-AVV-9/miRNA-1 NB was prepared by linking biotinylated-pAd-AVV-9/miRNA-1 to biotinylated NBs by the “biotin-avidin method” (11). The fusion of two different excitation spectra was observed under the laser confocal microscope. pAd-AAV-9/miRNA-1 NB appeared yellow under the microscope, while the NBs were still red, indicating that the pAd-AAV-9/miRNA-1 was successfully attached to the surface of the NBs. The zeta analyzer measured the average diameter of pAd-AAV-9/miRNA-1 NB to be 500 nm, which was in line with the nanoscale size and could pass through the blood vessel wall.

This study demonstrated that the constructed pAd-AAV-9/miRNA-1 NB were in line with the characteristics of viral load saturation. The virus-carrying rate of nanobubbles gradually increased. After reaching the peak concentration, the virus-carrying rate decreased, which was related to the saturation of avidin-binding sites. Therefore, the optimal viral load of pAd-AAV-9/miRNA-1 NB was 5 μL and the optimal ratio of pAd-AAV-9/miRNA-1 viral to NBs was 1: 20, thereby achieving the optimal targeting performance and also improving the use of the virus at the same time.

Contrast-enhanced ultrasound imaging can not only target and visualize the target tissue but also help in the quantitative analysis of the signal intensity. The results of *in vitro* contrast-enhanced ultrasound experiments in this study showed that NBs and pAd-AAV-9/miRNA-1 NB produced contrast-enhanced signals in the imaging matrix model, and both reached the maximum strength signal intensity after 30-s injection. It showed that the pAd-AAV-9/miRNA-1 NB had good imaging ability. In addition, Zhang et al. (12) confirmed that NB_AMH_ could early and dynamically monitor the ovarian survival rate after transplantation through the quantitative analysis of NB_AMH_ angiographic signal intensity in ovarian tissue before and after transplantation. Therefore, the *in vivo* myocardium-targeted ultrasound imaging results in this study showed that both pAd-AAV-9/miRNA-1 NB and NBs could rapidly visualize myocardial tissue within 30 s after injection via the tail vein. After 30 s, the signal intensity of pAd-AAV-9/miRNA-1 NB in myocardial tissue continued to increase, but the NBs began to decline after reaching the peak signal intensity. The pAd-AAV-9/miRNA-1 NB reached the peak signal intensity in 1 min and continued to enhance imaging for up to 15 min. The quantitative analysis of the contrast signal intensity of pAd-AAV-9/miRNA-1 NB in the myocardial tissue further confirmed that it could target and visualize myocardial tissue and achieve ultrasound image visualization of the target tissue. However, NBs are rapidly metabolized in the blood circulation because they have no target-binding ability. Therefore, the gene delivery system carried by nanobubbles and mediated by targeted functional carriers constructed in this study achieved the development of target tissues and enabled real-time ultrasound monitoring and tracking of target genes.

The results of tissue fluorescence imaging in this study showed the distribution of the pAd-AAV-9/miRNA-1 NB in the whole body of animals. We observed that NBs and pAd-AAV-9/miRNA-1 NB produced Dil uptake fluorescence signal intensity in the heart, lung, spleen, liver, and kidney *ex vivo* during the same time period. With the prolongation of time, the fluorescence signal intensity of Dil uptake in the heart of SD rats in the pAd-AAV-9/miRNA-1 NB group continued to increase for more than 35 min, while the attenuation trend appeared in the lung, spleen, liver, and kidney. However, within 35 min, the expression level of Dil uptake fluorescence signal intensity in the heart of pAd-AAV-9/miRNA-1NB group was relatively low, while the expression level of Dil uptake fluorescence signal intensity in the spleen and kidney was relatively high, which may be due to several factors, such as differences in receptor expression and clearance mechanisms, insufficient observation time or differences in microenvironmental conditions in the kidneys and spleen. Thus, under the same duration of 35 min, we also quantitatively detected the expression level of miRNA-1 in vital organs *ex vivo* at different time points by Q-PCR. The results showed that with the extension of time, the expression level of miRNA-1 in the heart continued to increase, and the expression level of miRNA-1 in other vital organs continued to decrease. The expression level of miRNA-1 in the heart at each time point was significantly higher than that in other vital organs. The relatively high expression level of Dil uptake fluorescence signal intensity in the spleen and kidney is not parallel to the expression level of miRNA-1 in the kidney and spleen, indicating that overexpression of pAd-AAV-9/miRNA-1 NB has cardiac specificity and achieved targeted accumulation to the myocardial tissue. At the same time, We found that Dil uptake fluorescence signal intensity of pAd-AAV-9/miRNA-1NB also accumulated in the lung within the first 15 min, but as the metabolic time prolongs, the Dil uptake fluorescence signal intensity of pAd-AAV-9/miRNA-1 NB rapidly decreases in the lungs after 15 min, Indicating that the accumulation of Dil uptake fluorescence signal intensity of pAd-AAV-9/miRNA-1 NB in the lungs is different from its targeted accumulation in myocardial tissue, therefore it was in line with the metabolism characteristics of lipid ultrasound nanobubble in blood circulation. After achieving accumulation in targeted tissues, it was mainly metabolized through the pulmonary circulation (13, 14).

The targeting vector (AAV9.IGF-1Ea) constructed by Enrique et al. (15) and the targeting liposome miR-21 (cT-21-LIPs) constructed by Minghui Li et al. (16) were used as delivery vehicles. The combination of the vector and the target gene could provide structural protection for the packaged gene, but could not realize the visualization of the overall vector. The targeted functional vector pAd-AVV-9/miRNA-1 constructed in this study was composed of a protein capsid and a small single-stranded DNA genome (8, 17). Its wrapping of protein capsid enabled shell-like structural protection for miRNA-1. At the same time, Q-PCR results showed that miRNA-1 was expressed in the myocardium of pAd-AAV-9/miRNA-1 NB group and pAd-AAV-9/miRNA-1 NB + UTMD group. In addition, the expression level of miRNA-1 in myocardium was significantly increased after targeted site blasting in pAd-AAV-9/miRNA-1 NB + UTMD group. This result confirmed that the pAd-AAV-9/miRNA-1 NB successfully targeted myocardial tissue through the cardiotropic effect of recombinant type 9 adenovirus (9), and applied UTMD technology (18) to achieve targeted targeted blasting of nanobubbles, release the carrier gene, efficiently transfect the target tissue under the ultrasound vision (19, 20).

The pAd-AAV-9/miRNA-1 NB served as the novel gene delivery system. The CCK-8 experiment confirmed that under the intervention of 1 × 10^8^/mL highest concentration gradient of pAd-AAV-9/miRNA-1 NB, the cell proliferation ratio still exceeded 90%. The preparation material of pAd-AAV-9/miRNA-1 NB has no toxic effect on cardiomyocytes.

Our research has several limitations. Firstly, this is only a preliminary feasibility study, subsequent research will further explore the targeted transfection rate of pAd-AAV-9/miRNA-1 NB to myocardial cell, in order to achieve the optimal effect of miRNA-1 targeted transfection to myocardial cell. Secondly, we failed to use Fluorescence *in situ* hybridization (FISH) staining of myocardial slices or immunohistochemistry of sections of rat hearts to accurately locate and identify the target cell population of miRNA-1 in the organ. In subsequent experiments, we will strive to overcome technical limitations and achieve a general understanding of uptake、expression and function of miRNA-1 targeted transported by pAd-AAV-9/miRNA-1 NB in related cell types *in vivo*.

## 5. Conclusion

In this study, a pseudo-endogenous microRNA-targeted myocardial pAd-AAV-9/miRNA-1 NB was successfully constructed. By simulating the existence of endogenous miRNAs in the circulation *in vivo*, the naked miRNA-1 synthesized *in vitro* was successfully transported to the myocardial tissue, and the targeted development of the myocardial tissue was achieved.

## Data availability statement

The raw data supporting the conclusions of this article will be made available by the authors, without undue reservation.

## Ethics statement

The animal studies were approved by ethics review approval number (K202202-12). The studies were conducted in accordance with the local legislation and institutional requirements. Written informed consent was obtained from the owners for the participation of their animals in this study.

## Author contributions

AA contributed to the study design, data collection, statistical analysis, visualization, writing and revised original draft. YW, JD, and LT revised the manuscript and performed the experiments. LG and YM led the study and provided scientific supervision. All authors contributed to the article and approved the submitted version.

## Funding

This research was supported by the Natural Science Foundation of Xinjiang Uygur Autonomous Region (Grant No. XJEDU2018I013), “State Key Laboratory of Pathogenesis, Prevention and Treatment of High Incidence Diseases in Central Asia Fund” (Grant No. SKL-HIDCA-2021-XXG6), the Regional Cooperative Innovation Program of Autonomous Region (Science and Technology Aid Xinjiang Program, Grant No. 2018E02062), and the Xinjiang Key Laboratory of Ultrasound Medicine.

## Conflict of interest

The authors declare that the research was conducted in the absence of any commercial or financial relationships that could be construed as a potential conflict of interest.

## Publisher’s note

All claims expressed in this article are solely those of the authors and do not necessarily represent those of their affiliated organizations, or those of the publisher, the editors and the reviewers. Any product that may be evaluated in this article, or claim that may be made by its manufacturer, is not guaranteed or endorsed by the publisher.
