# Supplementary material for: Functional evaluation of constructed pseudo-endogenous microRNA-targeted myocardial ultrasound nanobubble
Source: Front Med (Lausanne). 2023 Sep 22;10:1136304. doi: 10.3389/fmed.2023.1136304 (PMC10556731; doi:10.3389/fmed.2023.1136304)
Supplement: Supplementary file 1 [file Data_Sheet_1.PDF]

Supplementary Material

Functional evaluation of constructed pseudo-endogenous microRNA-targeted myocardial ultrasound nanobubble

Ailifeire Ainiwan [1], Yuanyuan Wei [1] ,Jing Dou [1],Lingpeng Tang [1],Yuming Mu [1]and Lina Guan [1,*]

[1]Department of Echocardiography, The First Affiliated Hospital of Xinjiang Medical University, Xinjiang Medical University,Urumqi, China

*** Correspondence:** Lina Guan;sanjin_lsx@163.com

Supplementary Table 1. Primers sequence

Primer Information	Primer	Primer sequence (5'-3)
	U6-S	CTCGCTTCGGCAGCACA
	U6-A	AACGCTTCACGAATTTGCGT
MIMAT0003162	rno-miR-1-5p-RT	CTCAACTGGTGTCGTGGAGTCGGCAATTCAGTTGAGGGGTACAT
	rno-miR-1-5p-S	ACACTCCAGCTGGGGCACATACTTCTTTAT
	Universal Primer-A	TGGTGTCGTGGAGTCG
